# How does tuning the van der Waals bonding strength affect adsorbate structure?

**DOI:** 10.1039/d2cp03468a

**Published:** 2022-11-10

**Authors:** Philipp Maier, Neubi F. Xavier, Chris L. Truscott, Thomas Hansen, Peter Fouquet, Marco Sacchi, Anton Tamtögl

**Affiliations:** Institute of Experimental Physics, Graz University of Technology 8010 Graz Austria philipp.maier@tugraz.at; Department of Chemistry, University of Surrey Guildford GU2 7XH UK; Department of Chemistry, University of Cambridge Lensfield Road Cambridge CB2 1EW UK; Institut Laue-Langevin 71 Avenue des Martyrs 38000 Grenoble France

## Abstract

Organic molecular thin-films are employed for manufacturing a wide variety of electronic devices, including memory devices and transistors. A precise description of the atomic-scale interactions in aromatic carbon systems is of paramount importance for the design of organic thin-films and carbon-based nanomaterials. Here we investigate the binding and structure of pyrazine on graphite with neutron diffraction and spin-echo measurements. Diffraction data of the ordered phase of deuterated pyrazine, (C_4_D_4_N_2_), adsorbed on the graphite (0001) basal plane surface are compared to scattering simulations and complemented by van der Waals corrected density functional theory calculations. The lattice constant of pyrazine on graphite is found to be (6.06 ± 0.02) Å. Compared to benzene (C_6_D_6_) adsorption on graphite, the pyrazine overlayer appears to be much more thermodynamically stable, up to 320 K, and continues in layer-by-layer growth. Both findings suggest a direct correlation between the intensity of van der Waals bonding and the stability of the self-assembled overlayer because the nitrogen atoms in the six-membered ring of pyrazine increase the van der Waals bonding in comparison to benzene, which only contains carbon atoms.

## Abstract 2

The binding and structure of an ordered pyrazine overlayer on graphite are studied with neutron scattering and DFT. We find increased van der Waals bonding and stability of the overlayer compared to benzene, due to the nitrogen atoms in the ring.

## Graphical abstract

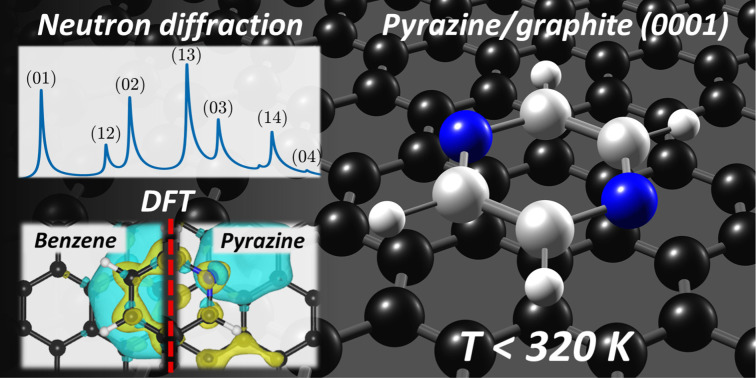

## 1 Introduction

In the last few decades, the growth of organic self-assembled overlayers on surfaces has attracted much interest due to their applicability in many technological fields such as opto-electronics and semiconductor devices.^1^ The assembly and stability of organic thin-films on surfaces is largely determined by their structural properties such as alignment, molecule–substrate interactions and defects. Moreover, an exact description of the interactions in aromatic carbon systems is of paramount importance for the design of carbon-based nanomaterials.^2^ Here we address the question of how the van der Waals bonding strength upon adsorption of aromatic organic compounds on graphite influences the adsorbate structure, thermal stability and growth mode.

**Fig. 1:**
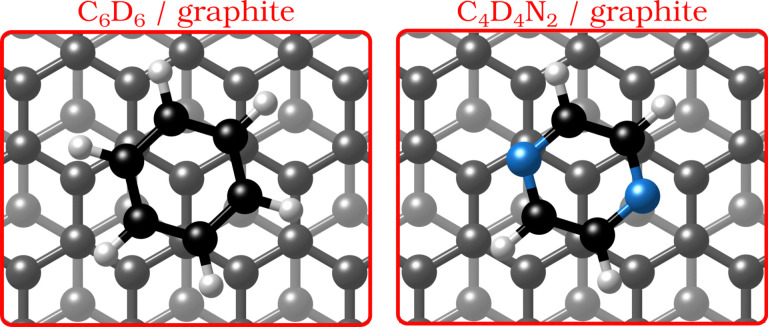
Graphical impression of the heterocyclic organic molecules benzene (C_6_D_6_) and pyrazine (C_4_D_4_N_2_) adsorbed on graphite.

By replacing some of the C atoms in benzene (C_6_H_6_) with N atoms (see Fig. 1), as done in the described measurements, a direct comparison can be made between the surface structure and dynamics of aromatics that have a similar ring geometry, but different aromaticity and electronic structure. The introduction of N atoms into the ring reduces the electronic density around the carbon atoms of the molecule and decreases the repulsion between the π-orbitals of the ring and the substrate, giving rise to stronger bonding to the substrate, because the intensity of the van der Waals (vdW) interactions is tuned by the polarisability and electrophilic character of the π-systems of the molecules interacting with the π-system of graphite.^3–5^

We address the question using measurements of the structure of deuterated pyrazine (C_4_D_4_N_2_) adsorbed on exfoliated graphite and assessing its temperature and coverage dependence in comparison with benzene. There already exist some studies on the structure and dynamics of hydrocarbons and small molecules on graphite^6–9^ and in particular for benzene, very detailed spectroscopy data have become available in recent years.^8–10^

Benzene adsorbs in a flat (face–face) configuration on graphite and follows Brownian diffusion on this substrate as shown in previous neutron scattering experiments.^8,9^ Contrary to benzene, the self-assembly behaviour of nitrogen-containing heterocyclic compounds such as pyrazine (C_4_H_4_N_2_, see Fig. 1) and triazine (C_3_H_3_N_3_)^11^ on carbon surfaces is largely unknown, although previous theoretical studies confirm that, similar to benzene, these N-containing molecules adsorb with a horizontal configuration at a distance of 3.00–3.21 Å.^3^ Indeed, as shown experimentally, the adsorption geometry of all three molecules on single-crystal metal substrates is in a flat configuration.^12–14^ Moreover, scanning tunnelling microscopy (STM) measurements demonstrated that 1,3,5-triazine (*s*-triazine) adsorbs parallell on highly oriented pyrolytic graphite (HOPG).^13^ Hence, while the adsorption geometry of the molecules remains the same for all three adsorbates, the intensity of the vdW-interactions will be tuned by the number of nitrogen atoms in the ring.^3–5^

The adsorption and diffusion of heterocyclic aromatic compounds such as pyrazine and triazine are also interesting for the modification/doping of graphene and graphitic substrates as well as for gas sensing purposes.^5,15,16^ It is known that the electronic properties of graphene can be tuned by noncovalent modification *via* adsorption of heterocyclic aromatic molecules. Reversible adsorption is an effective route to chemical doping of graphene and it has been shown that the adsorption of molecules such as triazine, pyrazine and borazine on graphene results in a widening of the band gap,^5,17^*i.e.* the chemical doping of graphene depends strongly on the electrophilic character of the dopants. Finally, the adsorption and dynamical properties of aromatic hydrocarbons have recently also attracted attention in the context of astrochemical processes occurring on cosmic dust grains.^18–21^

Here, we present data from neutron scattering experiments of deuterated pyrazine (C_4_D_4_N_2_) adsorbed on the (0001) basal plane surface of exfoliated graphite. We use deuterium-substituted pyrazine, C_4_D_4_N_2_, instead of C_4_H_4_N_2_ because hydrogen scatters neutrons predominately incoherently and, hence, contributes very little to the diffraction peaks.^10^ From temperature-dependent neutron diffraction measurements below and above monolayer coverage, we observe the formation of an ordered hexagonal overlayer of pyrazine on graphite which starts to melt at higher temperatures. These findings are further supported by neutron spin-echo (NSE) measurements and additional insights into the adsorption mechanism are provided by van der Waals corrected density functional theory (DFT) calculations.

## 2 Experimental and computational details

### 2.1 Sample preparation

Since the surface signal of hydrocarbon molecules adsorbed on graphite is weak one has to use high surface density substrates in neutron and X-ray experiments.^10^ As the substrate we used exfoliated compressed graphite, *Papyex*, which exhibits an effective surface area of about 20 m^2^ g^−1^ and retains a sufficiently low defect density.^22,23^ Due to its highly specific adsorption surface area it is widely used for adsorption measurements. We further exploit the fact that exfoliated graphite samples exhibit preferential orientation of the basal plane surfaces, which we oriented parallel to the scattering plane of the neutrons. Each sample was prepared with 13–14 g of Papyex exfoliated graphite of grade N998 (>99.8% C, Carbone Lorraine, Gennevilliers, France). The prepared exfoliated graphite disks were heated to 973 K under vacuum before transferring them into a cylindrical aluminium sample cartridge. The amount of powder C_4_D_4_N_2_ required to reach the corresponding monolayer (ML) coverage, was weighed using a fine balance and then added to the graphite disks. The aluminium sample holders were hermetically sealed using a lid with a steel knife-edge. The samples were then heated in an evacuated furnace to 380 K to sublimate the powder and promote its adsorption in the whole volume of the sample.

### 2.2 Diffraction measurements

**Fig. 2:**
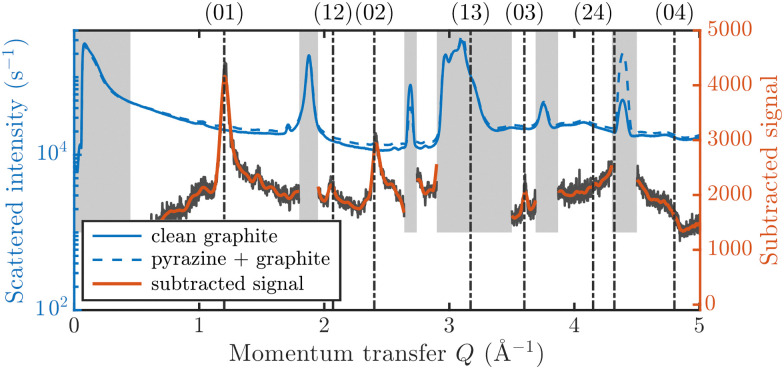
Diffraction data at 275 K for the pristine graphite sample and a coverage of 0.9 ML of C_4_D_4_N_2_ together with the subtracted data (300 K). Regions with a strong graphite signal are indicated by a grey patch and are omitted in the subsequent analysis.

Neutron diffraction measurements were carried out using the highintensity powder diffractometer D20 at Institut Laue-Langevin (ILL), Grenoble, France, using a wavelength of *λ* = 2.41 Å. The data was taken in a range of momentum transfers *Q* = |**Q**| = |**k**_f_ − **k**_i_| = [0.7–5.1] Å^−1^, where **k**_i_ and **k**_f_ are the neutron wave vectors before and after scattering from the sample, respectively. The wave vector transfer **Q** can be calculated from the diffraction angle between the incoming and scattered beam *ϑ*:1
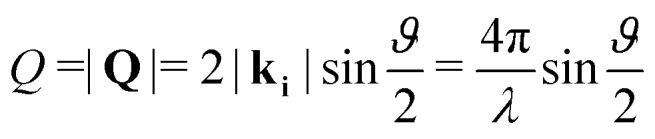
Measurements were performed at relative surface coverages of 0.9 and 1.5 ML, respectively, and at temperatures in the range 50 to 350 K. The temperature was controlled using a standard liquid helium cryostat (“orange” cryostat). Additional diffraction measurements of the clean graphite sample were obtained at 50 K, 300 K and 350 K. The graphite substrate and its orientation remained the same throughout all measurements. Subsequently, the diffraction scans for pristine graphite were subtracted from the diffraction data of C_4_D_4_N_2_/graphite at the same temperature (see Fig. 2). Since we did not perform measurements of the clean graphite substrate at temperatures of 100 K, 250 K, 275 K and 320 K we subtracted in these cases the clean graphite data at the nearest available temperature (*e.g.* from the pyrazine data measured at 275 K we subtracted the clean graphite spectrum at 300 K). Five regions in our data show very strong signals from the graphite substrate that masks the signal from the pyrazine adsorbate and makes a meaningful interpretation of the pyrazine signal impossible. These regions, as well as the low *Q* region, are not shown in Fig. 2 and are excluded from further data analysis.

### 2.3 Neutron spin-echo measurements

In addition to the diffraction scans a series of neutron spin-echo (NSE) measurements were performed. In NSE spectroscopy, the Larmor precession of the neutron spin in a magnetic field is exploited to decouple the instrumental resolution from the wavelength distribution of the neutron beam.^24,25^ This results in a much higher spatial and temporal resolution (compared to time-of-flight measurements) since only the energy exchange during scattering is measured rather than the absolute scattered energy distribution.^24,26^ The quantity that is measured is the real part of the normalised intermediate scattering function *I*(*Q*,*t*)/*I*(*Q*,0), which is related to the scattering function *S*(*Q*,*ω*) *via* a Fourier transform in time. Here, *ħω* is the energy transfer to the sample and *t* is the (spin-echo) time. Since *I*(*Q*,*t*) describes the correlation in reciprocal space and real-time, spin-echo spectroscopy provides a measure of the decay of structural correlation with time, on the length-scale and direction given by *Q*.^27,28^ A quantum mechanical description of NSE spectroscopy illustrates the origin of the correlation measurement.^26^ Due to its ability to measure structural correlation over time, spin-echo spectroscopy is perfectly suited to investigate temperature-dependent structural changes and the dynamics of hydrogen-containing molecules at interfaces.^10,27–29^

The neutron spin-echo experiments were performed on the NSE spectrometer IN11 at ILL using the high signal set-up IN11C, which uses a 30-degree detector bank.^30^ The NSE measurements were performed at a coverage of 0.5 ML for various temperatures ranging from *T* = 100 K to *T* = 400 K. A wavelength of *λ* = 5.5 Å was used for the maximum signal and the experiment covered a range of momentum transfers of *Q* = [0.2–0.7] Å^−1^. A standard procedure for normalising the spectra was used: A spectrum at the cryostat base temperature of 1.5 K was obtained *in situ*. At this temperature, it is reasonable to assume that the system is static in the dynamic window of the spectrometer.^10^ For normalisation, all NSE data were then divided by this spectrum.

### 2.4 Computational details

DFT calculations were performed using the plane wave periodic boundary condition code CASTEP.^31^ We used the Perdew Burke Ernzerhof^32^ exchange-correlation functional, with the dispersion force corrections developed by Tkatchenko and Scheffler (TS method)^33^ for the calculations presented in this work. The choice of (TS) method is motivated by our long experience with the correction scheme for studies on graphene and graphite.^34–36^ We consistently found that the TS method offers a good compromise between accuracy and effectiveness, being in general more accurate than other more semi-empirical corrections and much less computationally demanding than many-body correction schemes.^34,37^ The plane wave basis set was truncated to a kinetic energy cutoff of 400 eV. The adsorbate system was modelled using a (9 × 9) graphene unit cell composed of a three-layer graphene sheet and a vacuum spacing of 20 Å above the graphite surface in order to avoid interactions with the periodically repeated supercells. As an ionic constraint, all the carbon atoms were fixed in the graphene substrate during the calculations. All the calculations used Vanderbilt Ultrasoft Pseudopotentials.^38^ The electron energy was converged up to a tolerance of 1 × 10^−8^ eV while the force tolerance for the geometrical optimisations was 0.01 eV Å^−1^.

## 3 Results and discussion

### 3.1 Adsorbate structure

**Fig. 3:**
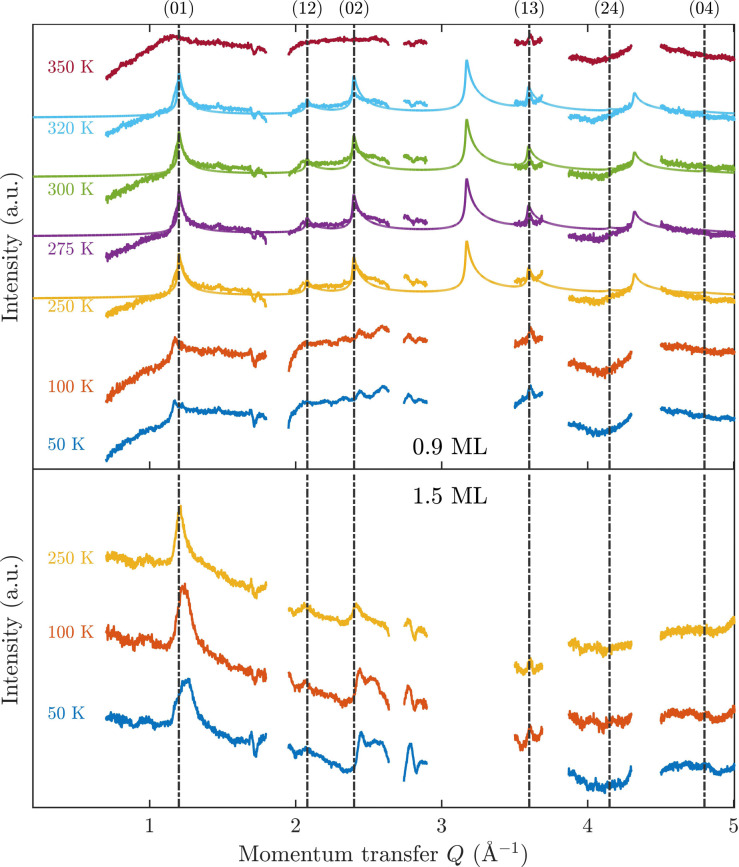
Subtracted diffraction scans of C_4_D_4_N_2_ for various temperatures at a coverage of 0.9 ML (upper panel) and 1.5 ML (lower panel). The vertical dashed lines show the position of the predicted peak positions (Table 1), while several calculated diffraction scans are superimposed as solid lines in the upper panel. The diffraction patterns at both coverages are consistent with a hexagonal superstructure of C_4_D_4_N_2_ adsorbed in a flat face-to-face configuration (see text). Only at temperatures above 320 K does the diffraction pattern disappear pointing towards a gradual melting.

Fig. 3 displays an overview of the coverage and temperature-dependent diffraction scans of pyrazine/graphite where the measurement data of the clean graphite surface have been subtracted from the deuterated pyrazine data as described above. The vertical dashed lines show the position of the theoretically predicted diffraction peaks (Table 1), which will be discussed in the following. The two coverage regimes (0.9 ML and 1.5 ML) look very similar to each other (at the same temperatures) apart from the subtraction being more problematic for the higher coverage data. In both regimes, the first and second order diffraction peaks (01) and (02) are clearly visible whereas the other theoretically predicted peak positions are either rather weak or shadowed by the substrate (graphite) diffraction peaks. The pyrazine peaks remain approximately at the same position, merely the peaks at a higher coverage and *T* = 50 K are slightly shifted with respect to the predicted position indicating a small low-temperature contraction of the overlayer. All peaks are characterised by an asymmetrical shape that is typical for diffraction patterns of two-dimensional poly-crystalline systems.^39,40^ The origin of these asymmetric shapes is the random orientation of crystallographic domains, where the normal of the domain remains parallel to the surface normal.^10^

**Table 1:** Summary of simulated and experimentally determined diffraction peak positions from C_4_D_4_N_2_/graphite, for different diffraction peak orders. The experimental values are for the 0.9 monolayer sample and at temperatures below *T* = 320 K

Order	Simulated	Experiment
	*Q* (Å^−1^)	*Q* (Å^−1^)
(0,1)	1.20	1.20
(1,2)	2.08	2.06
(0,2)	2.40	2.40
(1,3)	3.17	Hidden
(0,3)	3.60	3.61
(2,4)	4.15	Weak
(1,4)	4.32	Hidden
(0,4)	4.80	Weak

For a more detailed analysis of the structures we have simulated neutron diffraction patterns of a flat-lying monolayer using the software package nxpattern, which allowed us to adjust a number of parameters manually.^41^ The results of this manual structural refinement are superimposed onto the diffraction signal in the upper panel of Fig. 3, while the theoretical peak positions are given in Table 1. The positions of the atoms in the deuterated pyrazine molecule were based on crystallographic data from X-ray diffraction.^42^

We have then adjusted the lattice parameters and found clear consistency with the model of a monolayer of densely packed flat-lying pyrazine molecules. In this model, the pyrazine molecules exhibit a hexagonal unit cell with *a* = *b* = (6.06 ± 0.02) Å. According to the intensity ratios in the diffraction pattern, each C_4_D_4_N_2_ molecule is rotated by ≈40° around the *C*_2_ axis (see Fig. 5(a)) with respect to the graphite (0001) lattice. In the next step we have simulated the results for out-of-plane tilting of the molecules where we can exclude any tilt of more than 10 degrees, due to the subsequent mismatch between the calculated and measured diffraction spectra thus confirming the flat adsorption geometry.

**Fig. 4:**
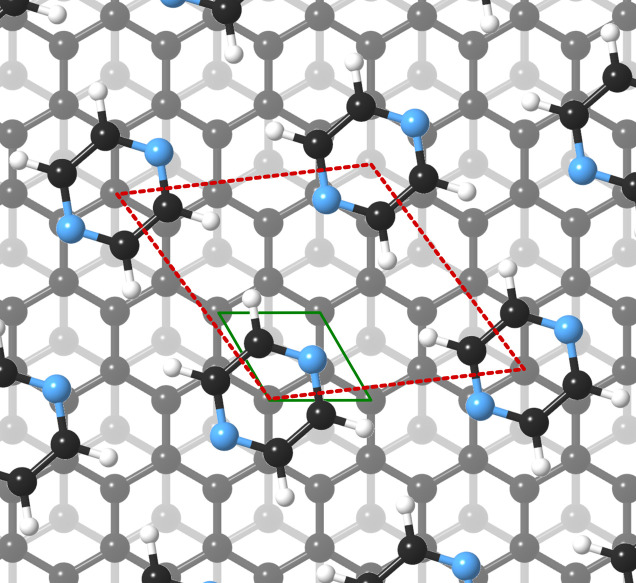
Proposed structure of a pyrazine (C_4_H_4_N_2_) monolayer adsorbed on graphite. Placement of the experimentally determined periodic pyrazine overlayer on graphite (0001) corresponds to an incommensurate (2.52 × 2.52)*R*6.6° superstructure as illustrated by the red dashed rhombus.

For this kind of regular arrangement of pyrazine molecules, a possible arrangement on the graphite (0001) surface is an incommensurate (2.52 × 2.52)*R*6.6° superstructure as illustrated by the red dashed rhombus in Fig. 4. For comparison, the pyrazine bulk crystal structure is completely different to the flat monolayer and given by an orthorhombic crystal system with two molecules in the unit cell, space group *Pnnm*, with cell constants *a* = 9.325 Å, *b* = 5.850 Å and *c* = 3.733 Å.^42,43^ In the bulk crystal structure, the molecules are interconnected through hydrogen bonds. The two shortest contact distances C–H⋯H–C and N⋯H–C are almost orthogonal with the closest bonding distance being the N⋯H bond at 2.607 Å.^44^

**Fig. 5:**
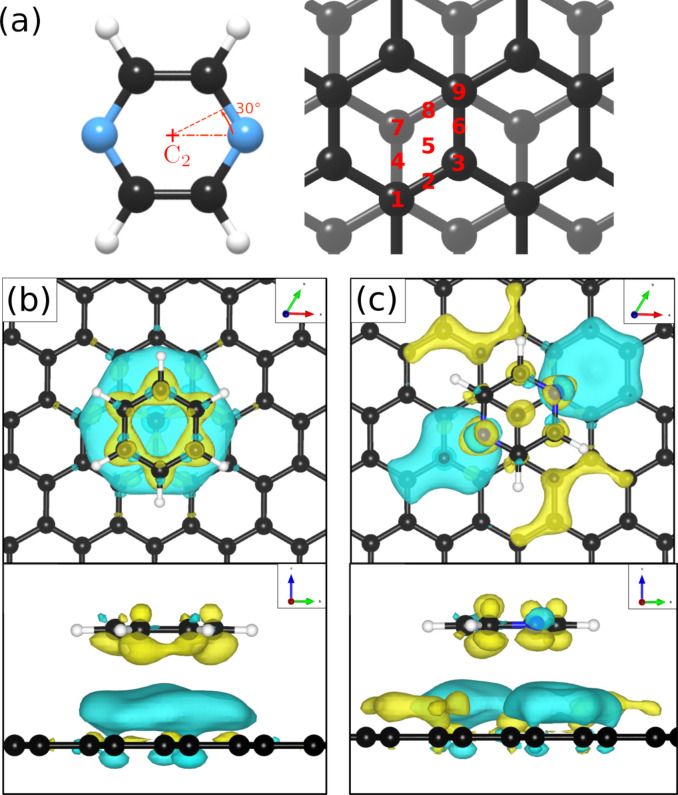
(a) Different adsorption sites and rotational angles of the C_4_H_4_N_2_ molecule probed in the DFT calculations. (b) and (c) show a top and side view of the energetically most favourable adsorption geometry of C_6_H_6_ and C_4_H_4_N_2_ on graphite(0001), respectively, together with the charge density distribution based on vdW corrected DFT calculations. Charge accumulation and charge depletion with respect to isolated benzene and pyrazine, are shown in yellow and blue, respectively with the isosurface cut-off set to 0.01 e Å^−3^.

Our results are similar to the findings of Bahn *et al.* who studied the adsorption mechanism of deuterated benzene on graphite based on neutron scattering.^10^ According to Bahn *et al.* benzene adsorbs on graphite (0001) in a commensurate 
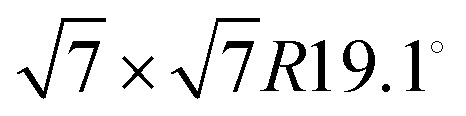
 structure with the hydrogen atoms being turned away from the carbon atom positions, *i.e.* completely different to its bulk structure.^45,46^ We also note that the unit cell of the pyrazine overlayer is smaller than the one found for benzene resulting in a denser packing of pyrazine on graphite, which is further discussed below.

It is also interesting to have a short discussion about the peak line shape which gives us additional information about the adsorbate structure. When neutrons are scattered in-plane from a two-dimensional layer of randomly orientated, ordered islands, the resulting diffraction peaks will drop sharply for lower angles and will decay as a Lorentzian for higher angles.^10,39^ The width of the peaks depends also on the experimental resolution Δ*Q*/*Q* and on the size of the islands. At lower coverage, the island size is smaller, which results in an increased width of the diffraction peaks in our diffraction measurements. We can also observe that the width of the peaks at a coverage of 1.5 ML increases with increasing temperature. This indicates that the size of the islands/clusters is reduced upon heating, in line with an increased mobility of the molecules. Lastly, it should also be mentioned that the peak height is significantly influenced by the thermal attenuation on the surface described by the Debye–Waller factor (DWF). The origin of this attenuation is the zero-point motion and thermal vibrations of the surface atoms causing a displacement **u** from their equilibrium positions, which leads to additional inelastic scattering of the incoming particles. The basic expression for the DWF can be derived by including this displacement of the scattering centre in the structure factor leading to an additional exponential prefactor, the Debye–Waller factor, given by DWF = 〈exp(*i***Q**·**u**)〉 where *Q* is the momentum transfer and 〈…〉 denotes the thermal average.^47^ Since the thermal displacements are random and uncorrelated with the direction of *Q* the leading order term in a series expansion of the exponential is of second-order in the dot product.^48^ Assuming a harmonic potential the expression for the DWF can be simplified to DWF = exp[−*Q*^2^〈*u*^2^〉/3] where 〈*u*^2^〉 is the mean square displacement of the atoms. With increasing temperature, thermal vibrations will give rise to inelastic scattering and an attenuation of the elastic peak intensities, while the peak shape remains unchanged.^47,49^ In addition, as can be seen from the DWF, the peak intensities also drop with increasing momentum transfer *Q* in Fig. 3.

A summary of the diffraction measurements and our simulation results from nxpattern are shown in Fig. 3. The agreement between the experimental data and the simulation confirms the formation of a hexagonal overlayer with *a* = (6.06 ± 0.02) Å, both for coverages of 0.9 ML (upper panel) and 1.5 ML (lower panel). The measured and simulated peak positions (black dashed lines) in the *Q*-range of our experiments are compared in Table 1. The position of the four “visible” experimental peaks is reproduced very well (within the second digit, similar to other neutron diffraction studies^10^) and small deviations can be explained by experimental uncertainties and the imperfect subtraction of the substrate signal. The overall agreement of the simulations with our experimental data is very good, especially in the low *Q* range while for higher values of the momentum transfer small deviations occur. It is interesting that the thermal attenuation behaviour that one would expect due to the Debye–Waller factor as explained above cannot be observed at lower temperatures. Furthermore, we notice that for the 0.9 ML regime the peaks arising from the ordered structure are vanishing at high temperatures (350 K), which is an indication of a melting process of the ordered structures and will be discussed in more detail below.

By looking at the high coverage (1.5 ML) data (lower panel in Fig. 3) one can clearly see the same diffraction peaks as for the 0.9 ML data, apart from the subtraction procedure becoming more problematic as mentioned above. Thus, the structure persists above the monolayer thickness and we, therefore, suggest that pyrazine is growing in a layer-by-layer fashion, at least for the first 2 layers. This finding is in contrast to the behaviour of benzene adsorbed on graphite, where at a coverage of 1.3 ML additional peaks appear which can only be explained by the growth of bulk crystalline benzene upon coverages exceeding the monolayer regime.^10^ The different behaviour of pyrazine compared to benzene can probably be attributed to an increased van der Waals bonding strength due to the introduction of nitrogen atoms with respect to the substrate as well as to a stronger intra-layer bonding of the pyrazine overlayer as further discussed in Section 3.3.

### 3.2 DFT calculations

To obtain additional theoretical insights into the adsorption mechanism of pyrazine on a graphite surface we complemented our experiments with van der Waals corrected density functional theory calculations for a single pyrazine molecule on a graphite surface. For this purpose, we have studied the adsorption of C_4_H_4_N_2_ on graphite for 9 different adsorption sites within the graphite unit cell that are labelled 1–9 in Fig. 5(a) and for two different rotations around the *C*_2_ axis. We calculated the adsorption energy of every single site for both rotations and the results of our calculations are summarised in Table 2. The energetically most favourable adsorption geometry of (C_4_H_4_N_2_) on graphite(0001) (*E*_a_ = −0.590 eV), based on vdW corrected DFT calculations, is for pyrazine in a 30° rotation over the graphite surface at position 1 (same as 9) and 3. The former is shown in Fig. 5(c) and further discussion of the electronic stability of pyrazine on graphite (0001) is made based on this geometry.

**Table 2:** The adsorption energy *E*_a_ and the energy difference Δ*E*_a_ relative to the most favourable adsorption site for C_4_H_4_N_2_ adsorption on graphite. The 9 different adsorption sites within the graphite unit cell (position 1–9 in Fig. 5(a)) refer to the *C*_2_ rotational axis through the centre of the ring

Pos.	30° rotation		0° rotation	
	*E*_a_ (eV)	Δ*E*_a_ (meV)	*E*_a_ (eV)	Δ*E*_a_ (meV)
1	−0.590	0	−0.580	10
2	−0.583	7	−0.577	13
3	−0.590	0	−0.581	9
4	−0.565	25	−0.568	22
5	−0.572	18	−0.572	18
6	−0.580	10	−0.574	16
7	−0.540	51	−0.545	45
8	−0.586	4	−0.573	17
9	−0.590	0	−0.580	10

In the aforementioned preferential adsorption sites (site 1 and 9 in Table 2) the pyrazine molecule is arranged in such a way that the position of the C and N atoms in the six-membered ring coincide with the second graphite layer. The least favourable position is with the C_4_H_4_N_2_ ring “sitting” directly on top of a graphite ring of the first layer. Otherwise, the energy differences between the sites, also with respect to in-plane rotations, are quite small (±50 meV).

We also note that in comparison to benzene, where DFT studies predict an adsorption energy of 0.495 eV^50^ according to vdW-DFT, pyrazine should in fact be bound slightly stronger (by about 95 meV) to graphite. Here, we carried out calculations with respect to the most favourable adsorption site of benzene on the graphite (0001) surface,^51^ obtaining roughly the same adsorption energy value, in comparison with C_4_H_4_N_2_, of −0.590 eV. It is noteworthy that the values reported were obtained for a low coverage scenario since calculations were made for a single adsorbate on a (9 × 9) supercell. Therefore, the impact of the higher coverage of benzene and pyrazine on the graphite (0001) surface was investigated by reducing the graphite supercell to (3 × 3) and calculating the values of *E*_a_. In this scenario, adsorption energy values of −0.680 eV and −0.648 eV are obtained for benzene and pyrazine, respectively. These results suggest that the higher stability of pyrazine, in comparison to benzene, is mostly related to stronger intermolecular interactions, due to the presence of N–H hydrogen bonds, present in the adsorbed C_4_H_4_N_2_ overlayer.

Investigations of the charge accumulation (yellow) and depletion (blue) were carried out for C_6_H_6_ and C_4_H_4_N_2_ above the graphite monolayer, as shown in Fig. 5(b) and (c), respectively. We observe a stronger dispersion interaction for benzene, evidenced by the charge depletion underneath the C_6_H_6_ ring.^37,52^ For pyrazine, the charge depletion region is strong in the interface region where the isolated electron pairs of nitrogen are located, which is confirmed by the partial charge of the nitrogen atoms of −0.390 *e*. The different topologies of the charge density regions between the benzene and pyrazine systems suggest a strong anisotropy for the diffusion and electronic friction for C_4_H_4_N_2_, in contrast to benzene diffusion.^8,9^

**Fig. 6:**
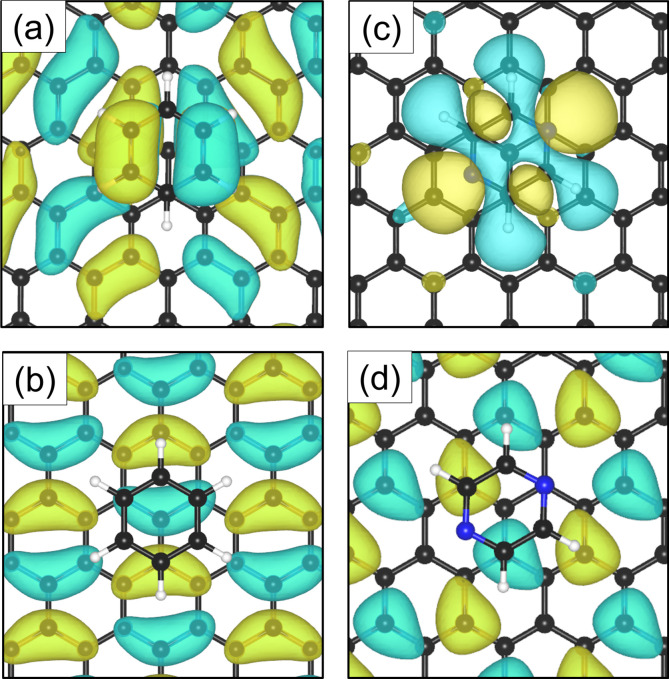
Top-view of the HOMO for benzene (a) and pyrazine (c) adsorbed on graphite. The LUMO orbitals are represented in (b) and (d) for benzene and pyrazine, respectively. In the case of C_6_H_6_, the π orbitals of the adsorbate and graphite (0001) surface contribute to the HOMO. For C_4_H_4_N_2_, a larger contribution to the HOMO comes from the adsorbed ring.

The electron density accumulation with respect to pyrazine is also observed in the analysis of the HOMO and LUMO orbitals, as shown in Fig. 6(c) and (d), respectively. The HOMO of the C_4_H_4_N_2_ system comprises a large contribution from the pyrazine orbitals, whereas the graphite (0001) surface orbitals are more present in the LUMO. With respect to the benzene system (Fig. 6(a) for the HOMO and Fig. 6(b) for the LUMO), the π orbitals from both the surface and the adsorbate contribute to the HOMO.

### 3.3 Temperature dependence and dynamics

In the following we consider the temperature dependence and thermal stability of the pyrazine overlayer, considering in particular the element of the increased vdW bonding strength with respect to benzene and the 2D self-assembly of other systems. Pyrazine can be seen as an interesting shorter analogue of 4,4-bipyridine.^53^ As shown recently for the latter, the influence of non-covalent interactions within an adsorbed adlayer, such as hydrogen bonding, gives rise to the formation of very stable solid adlayers even at temperatures above the bulk melting point.^53^

In the upper panel of Fig. 3 the diffraction measurements obtained at 0.9 ML coverage are shown for various temperatures. One can clearly see that the structure remains stable for a relatively large temperature range. At temperatures above 320 K the peaks are vanishing, which is an indication of a gradual melting process of the ordered structure. The melting temperature for the monolayer is roughly the same as the bulk melting temperature for pyrazine at 327 K. The melting temperature of the overlayer is thus much higher than for benzene adsorbed on the same graphite substrate, with benzene being liquid at room temperature and the gradual melting of the adsorbed structure being observed at 140 K.^10^

We thus conclude that the effect is two-fold: first, the introduction of N atoms into the benzene ring gives rise to stronger vdW interactions between the adsorbate and the substrate. Second, in analogy to 4,4-bipyridine adsorbed on graphite,^53^ we observe that the non-covalent interactions within the adsorbed monolayer lead to an increased melting temperature, even above the bulk melting point.

**Fig. 7:**
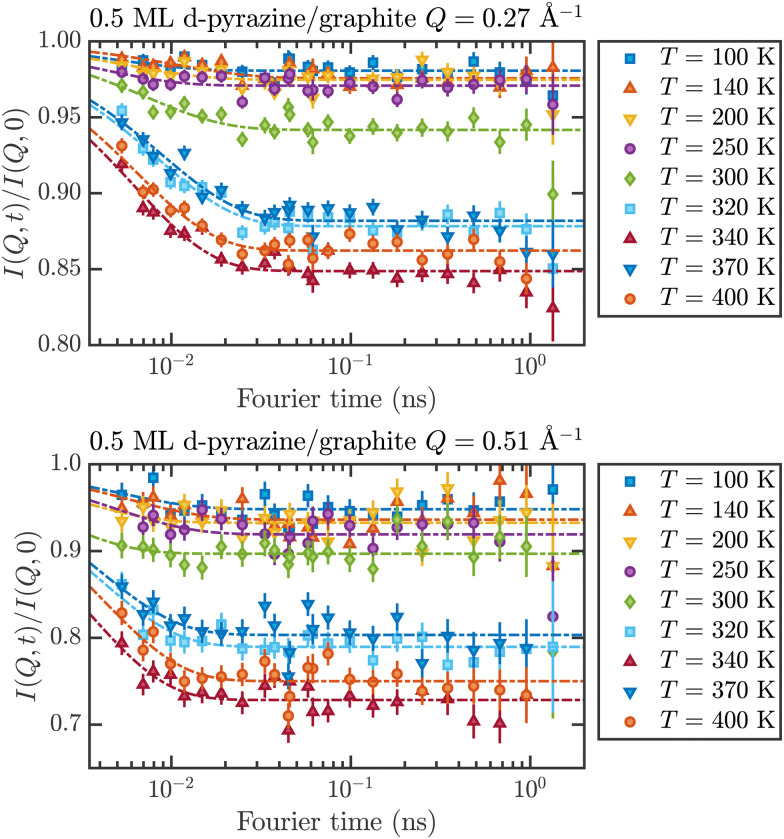
Neutron spin-echo spectra of a 0.5 ML pyrazine on graphite sample, measured at temperatures between 100 and 400 K at a momentum transfer of *Q* = 0.27 Å^−1^ (upper graph) and *Q* = 0.51 Å^−1^. The single spectra have been fitted by exponential decay functions according to eqn (2).

These temperature-dependent diffraction measurements are supported by neutron spin-echo data. NSE spectra have been recorded at a coverage of 0.5 ML at various temperatures between 100 K and 400 K. The obtained spectra are displayed in Fig. 7 at a momentum transfer of *Q* = 0.27 Å^−1^ and *Q* = 0.51 Å^−1^, respectively. The NSE dynamics measurements exhibit similarities with those of benzene on graphite in a similar coverage regime,^10^*i.e.* they can be described by an exponential decay that reaches a plateau value *A* at longer times:2
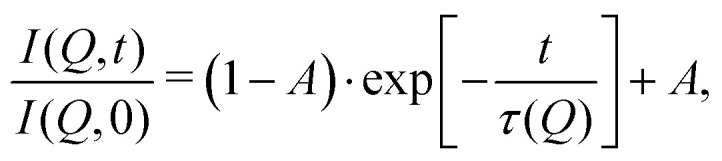
where *τ*(*Q*) is the decay time of the structural correlation function, the time at which *I*(*Q*,*t*) has decayed to 1/*e* of its value at *T* = 0, and in general is a function of the momentum transfer *Q*. The constant *A* is the level of the long time plateau and represents the scattering from the static part of the surface.

As explained in Section 2.3 the intermediate scattering function *I*(*Q*,*t*) provides a statistical description of the motion of the species from which the neutrons scatter and, hence, is a measure of the structural correlation.^24^ A static system maintains a perfect correlation with time, whereas changes in the atomic positions during the time window *t* lead to an overall decay in the level of *I*(*Q*,*t*). Simple models for surface diffusion give rise to an exponential decay as given by eqn (2).^24,54^ This corresponds to an exponential loss of correlation in the system. The decay or relaxation time *τ* contains a variety of information about the system. In general, the temperature dependence of 1/*τ* can be used to calculate activation barriers for the underlying motion, and the coverage dependence provides information about possible interactions between adsorbed species.^24^

**Fig. 8:**
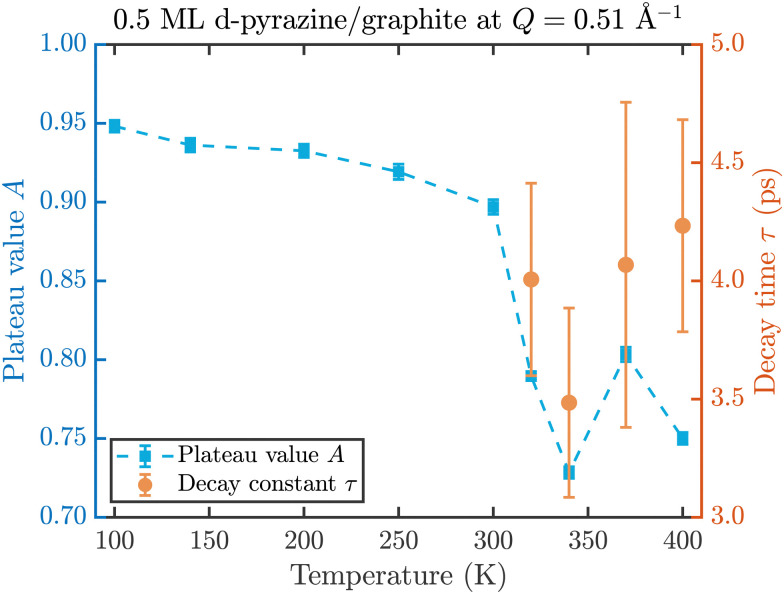
Temperature dependence of the plateau value *A* (blue squares) and the decay time *τ* (orange circles) at a momentum transfer of *Q* = 0.51 Å^−1^ from the fitted NSE data according to eqn (2). The strong drop of the static component *A* at 320 K confirms the melting temperature determined in the diffraction data while the dynamics occurs within a typical timescale of ≈4 ps.

The fit results for NSE spectra recorded at a momentum transfer of *Q* = 0.51 Å plotted against the temperature are shown in Fig. 8. The fitted values of *τ* (orange circles) are only displayed for temperatures higher than 300 K since the uncertainty is very large for lower temperatures. The decay constant *τ* for temperatures above 300 K (Fig. 8) illustrates that diffusion occurs in a timeframe of ≈4 ps at *Q* = 0.51 Å^−1^.

The clear drop of *A* (blue squares) at 320 K in Fig. 8 confirms that a strong dynamic motion starts to set in, since the plateau *A* represents scattering from the static fraction of the sample, that is the graphite substrate and immobile islands of pyrazine. The decreasing level of the plateau with temperature is another indication of the gradual melting of the ordered pyrazine layer and thus, it supports our interpretation of the vanishing diffraction peaks observed at high temperatures. We can therefore deduce that, for lower coverages, islands of deuterated pyrazine are formed on the graphite surface, which are arranged in a regular hexagonal overlayer with a unit cell length of *a* = (6.06 ± 0.02) Å. If the temperature is increased a successive melting of the ordered structure occurs.

The observed decay times of about 4 ps for *Q* = 0.51 Å^−1^ are equal within experimental uncertainty to the decay time observed for benzene diffusion on graphite at 140 K.^9^ In addition we find in both cases, pyrazine and benzene, a negligible temperature dependence of the decay constant, which indicates a low friction scenario in both cases. However, we observe a dramatic change in the melting behaviour of the adsorbed layers, with a much earlier melting for benzene and a complete desorption of benzene at around 150 K, whereas a substantial melting of pyrazine does not occur below 300 K.

Thus our dynamics measurements support the findings from the diffraction measurements, clearly indicating an increased bonding to the substrate as well as an increased intra-layer bonding. Moreover, the fact that the dynamics sets in at much higher temperatures and the negligible temperature dependence of the latter, suggests a very weak corrugation of the underlying potential energy surface over *k*_B_*T*. It also suggests a weak atomic-scale friction *η*, however, the latter would require further dynamics measurements over several length-scales *Q* as well as further analysis in light of a Langevin description of the dynamics.^55^ While the dissipative mechanisms *via* interactions with phonons and electrons in the substrate have been studied in light of vibrational relaxation^56^ and reactive scattering from surfaces,^57^ for friction in surface diffusion,^58^ there is no simple way to disentangle the phononic contribution from the electronic form of friction.^27,59^ Hence it would be particularly interesting to assess the influence of the vdW bonding strength with respect to the latter,^60^ as also outlined in Section 3.2.

### 3.4 Further comparison and discussion

Before concluding our study we return to our structural results and provide a further comparison with other systems and studies. The determined pyrazine/graphite structure in a hexagonal overlayer with *a* = (6.06 ± 0.02) Å means that the N⋯H distance between two neighbouring pyrazine molecules at 2.7 Å is almost the same as for bulk crystalline pyrazine despite the coplanar monolayer. In addition, the higher thermal stability and layer growth compared to benzene means that the pyrazine molecules on graphite exhibit a denser packing. The pyrazine structure is in fact quite similar to triazine upon adsorption on graphite as shown in a recent X-ray diffraction study of a crystalline monolayer of 1,3,5-triazine, where a hexagonal unit cell with a lattice parameter of 6.161 Å was found.^61^ However, as only one peak in the region of the diffraction pattern was observed it was impossible to assign relative peak intensities and thus atomic positions within the unit cell.^61^

The adlayer structure of pyridine, pyrazine and triazine on Cu(111) has also been analysed using STM measurements,^12^ where it was demonstrated that all three molecules exhibit the same flat (2 × 3) configuration. Hence, one can conclude that the packing density of C_4_H_4_N_2_ on Cu(111) is lower than on graphite, possibly caused by the different bonding mechanism to the metal substrate.

As mentioned above, pyrazine is a shorter analogue of 4,4-bipyridine. Following synchrotron X-ray diffraction the formation of crystalline monolayers of 4,4-bipyridine on graphite reveals a square unit cell with a lattice parameter *a* = 11.42 Å for higher temperatures and a rectangular unit cell with *a* = 11.26 Å and *b* = 11.45 Å with two atoms per unit cell.^53,62^ Considering the two atoms per unit cell and the fact that each molecule consists of two directly linked pyridine rings it can likely be translated to a similar if not even denser packing as for pyrazine/graphite. It further confirms the general trend of stronger bonding and denser packing upon the introduction of nitrogen atoms in the ring.

Finally, benzene can also be modified by replacing some of the hydrogen atoms with halogens (*e.g.* fluorine), resulting in so-called halobenzenes. The packing behaviour of the halobenzene 1,3,5-triodo-2,4,6-trifluorobenzene (TITFB) on graphite has been examined using X-ray diffraction, demonstrating the formation of a monolayer with an incommensurate hexagonal unit cell with a lattice parameter of 9.28 Å.^63^ Thus the replacement of H atoms in benzene with fluorine atoms has the opposite effect, *i.e*. an even larger spacing upon the individual TITFB molecules in the overlayer compared to benzene.

## 4 Summary and conclusion

In summary, we have studied the adsorption structure and thermal stability of deuterated pyrazine (C_4_D_4_N_2_) adsorbed on the (0001) basal plane surface of exfoliated graphite. Temperature-dependent diffraction measurements below and above monolayer coverage reveal an ordered hexagonal overlayer of pyrazine which gradually starts to melt at 320 K as further supported by neutron spin-echo measurements. Additional theoretical insight into the adsorption mechanism is provided by van der Waals corrected density functional theory (DFT) which predicts an adsorption energy of *E*_a_ = −0.572 eV in the most favourable adsorption geometry.

Compared to benzene (C_6_H_6_) self-assembly on graphite, the pyrazine overlayer appears to be much more thermodynamically stable and also persists above the monolayer thickness suggesting a layer-by-layer growth rather than the growth of a bulk crystalline structure as observed for benzene. Both findings suggest a direct correlation between the intensity of van der Waals bonding between the organic precursor and the substrate, due to the introduction of nitrogen atoms in the six-membered ring and the stability of the self-assembled overlayer. Moreover, the unit cell size of the pyrazine overlayer is very close to *s*-triazine on graphite, hence pyrazine exhibits a denser packing than benzene on graphite.

The NSE measurements show the same melting temperature as observed in the diffraction measurements and a structural decay constant, which is similar or equal to the one observed for benzene on graphite. In addition, we do not find a substantial temperature dependence of the decay constant and deduce that the increased van der Waals interaction between pyrazine and graphite strengthens the bonding to the substrate (higher melting and desorption temperature) without slowing the diffusive dynamics down. In light of these findings, we trust that a more detailed experimental and theoretical study of the system in comparison to the dynamics of benzene on graphite would be particularly interesting, in order to assess the atomic-scale friction and the influence of the vdW bonding on certain energy dissipation channels.

## Conflicts of interest

There are no conflicts to declare.

## Supplementary Material
